# U.S. Jails and fatal drug overdoses: patterns, predictors and the role of rehabilitative contexts

**DOI:** 10.1186/s40352-025-00365-1

**Published:** 2025-09-30

**Authors:** Victor St. John, Tasha Perdue, Jason Szkola, Mijin Kim, Katharine McGrath, Noa Glover, Josh Sugino

**Affiliations:** 1 The Ohio State University, Columbus, USA; 2 Rowan University, Glassboro, USA; 3 Independent Researcher, Chicago, USA; 4 Saint Louis University, St Louis, USA; 5 La Canada High School, La Canada Flintridge, USA; 6 Illinois State University, Illinois, USA

**Keywords:** Opioid-related deaths, Drug overdose, Rehabilitation, Punitive environments, Jails, Mortality, Corrections

## Abstract

Opioid-related fatalities in U.S. correctional facilities present a critical criminal justice and health challenge. This study examines predictors of drug- and opioid-related deaths among incarcerated individuals nationwide. In the main models, younger age increases overdose risk, females face higher odds of drug-related death than males, and shorter stays are linked to all drug-related deaths, while longer stays are associated with opioid fatalities. Geographic disparities emerge, with small metro and micropolitan areas showing higher drug death rates and large fringe metros showing significantly lower opioid death rates. Medium-security facilities and greater spatial distance from public transportation access points predict higher rates for both outcomes. Subgroup analyses reveal that conviction status predicts elevated drug-related mortality only among males and among individuals held longer than 17 days. Notably, over one-third of opioid-related deaths and more than half of other drug deaths occur within 24 h of incarceration, underscoring acute early-stage vulnerability. Findings reveal distinct and overlapping predictors shaped by both rehabilitative and punitive factors, informing policies and interventions to reduce overdose fatalities in jails.

## Introduction

Opioid-related deaths in U.S. jails represent a significant yet under-researched public health crisis. In 2020, the U.S. Bureau of Justice Statistics (BJS) estimated that the average daily population of persons held in jails across the country was 658,100, with 8.7 million annual admissions (Minton & Zeng, 2021). Jail has thus become a critical juncture where many encounter the criminal legal system. A substantial portion of this population struggles with substance use disorders: 53% have a substance use disorder (Compton et al., 2010) and 36% are drug dependent according to the Diagnostic and Statistical Manual of Mental Disorders (DSM-IV) criteria (Compton et al., 2010). The prevalence is even higher among sentenced populations, with approximately 63% meeting the DSM-IV criteria for drug dependence or abuse (Bronson et al., 2017:2020; Soloman et al., 2023). This paper focuses on a particularly urgent aspect of incarceration: opioid-related deaths.

Arguably, the most harmful outcome associated with time in jail is the loss of life (St. John, 2020), and fatalities attributed to substance use during incarceration are becoming increasingly prevalent. The Deaths in Custody Reporting Act (DCRA) mandates the collection of data on fatalities that occur while a person is under arrest, in transit to a correctional facility, or incarcerated, which is reported to the U.S. Attorney General and Department of Justice (Duwe, 2022). The latest DCRA reports indicate that in 2019, approximately 1,200 people died in local jails - the highest number since reporting began in 2000 (Carson, 2021). Notably, drug-related deaths peaked at an all-time high of 184 in 2019, marking a 397% increase from the 37 drug-related deaths reported in 2000 (Carson, 2021). This is the largest increase compared to other leading causes of death, such as suicide and medical illness in U.S. jails (Carson, 2021). However, DCRA reports do not disaggregate drug types, such as opioids, limiting an understanding of opioid-related deaths in correctional facilities. This omission is significant, given that opioids were responsible for 80,411 fatalities in the general population in 2021—a 17% increase from the 68,630 deaths in 2020 (National Institute on Drug Abuse, 2023). Without this data, policymakers, practitioners, advocates, and community-based organizations lack crucial information to develop interventions that could prevent opioid-related deaths in custody.

Despite growing attention to substance use and fatal outcomes, a critical gap remains in the understanding of opioid-related fatalities in correctional settings. This study addresses that gap by providing empirical insights that can inform policy, and correctional practices aimed at reducing these preventable deaths. The paper proceeds with: (1) an overview of deaths in custody and their relation to the rehabilitative ideal; (2) a review of literature on opioid-related deaths within correctional institutions and among formerly incarcerated persons; (3) an outline of the study’s research questions and hypotheses; (4) the study’s research design and methods; (5) the analytical approach and associated results; and (6) a discussion on the implications for research, practice, and policy.

## Rehabilitation in correctional institutions

Before exploring opioid-related fatalities in correctional settings, it is essential to understand the broader context of preventing deaths in these environments. Researchers working within the framework of criminal justice humanism argue that correctional institutions should prioritize addressing crime while ensuring the wellbeing of all individuals under their care (St. John et al., 2023). These institutions, including their physical environments, should be grounded in a moral and ethical responsibility to uphold the human rights and dignity of incarcerated individuals (Buchanan, 2001). Such expectations are enshrined in U.S. constitutional protections under the Eighth Amendment, which mandates the physical and psychological safety of persons in custody (Gorlin, 2009; Struve, 2012). These protections are also codified in various policies and practices, such as the 1980 U.S. Department of Justice standards on prison and jail operations, which emphasize the preservation of health and life in custody (Office of Justice Programs, 1980). The importance of protecting the health of incarcerated persons is further reflected in legislation such as the Death in Custody Reporting Program (2000) and the Death in Custody Reporting Act (2013, Public Law 113–242), which track fatalities in U.S. correctional facilities. Additionally, professional organizations such as the American Correctional Association, in partnership with the National Institute of Corrections and other bodies, have issued guidance on preventing harm, injury, and illness among incarcerated individuals (American Correctional Association, 2010; Solomon et al., 2023). These guidelines underscore the need for suicide prevention, mental health programs, detoxification protocols, and access to essential health services. Operationally, these understanding can fall under the commonly used term of rehabilitation^1^ – which is “a planned correctional intervention that targets for change internal and/or social criminogenic factors with the goal of reducing recidivism and, where possible, of improving other aspects of an offender’s life” (Cullen & Jonson, 2011). Cullen (2017) would simplify this as treatments that are planned; that address the drivers of crime among individuals; and that seek to improve the wellbeing of said persons, or transform individuals (Allen, 1959).

### The rehabilitative ideal

The rehabilitative ideal plays a foundational role in shaping both the ethical and operational framework of U.S. correctional institutions. At its core, the rehabilitative ideal posits that the primary function of the penal system is not only to punish but to rehabilitate individuals – transforming them into law-abiding members of society (Allen, 1959). This philosophy asserts that, given appropriate interventions and support, individuals who have engaged in criminal behavior can change and successfully reintegrate into their communities. The ideal is rooted in the belief that correctional institutions should prioritize health, safety, and rehabilitation over mere retribution (Rosazza, 2007). Additionally, the rehabilitative ideal is grounded in the understanding that criminal behavior often stems from underlying factors, such as substance use, mental health issues, or social disadvantages, which can be addressed through targeted interventions (i.e., the criminogenic factors Cullen and Jonson (2011) note). Research has consistently shown that addressing these factors, such as substance use (Best & Savic, 2020; Smith et al., 2022), mental health issues (Polaschek, 2019; Schaefer, 2018), or concentrated disadvantages (Jacobs & Skeem, 2021), can significantly reduce recidivism. A treatment-oriented, therapeutic model of corrections is argued to foster greater behavioral change and legal conformity (Cullen & Gilbert, 2012). One of the most widely accepted frameworks associated with the rehabilitative ideal is the risk-needs-responsivity (RNR) model. This model emphasizes the importance of identifying individual risks and needs and tailoring interventions to facilitate rehabilitation. The RNR model suggests that responsiveness to individual circumstances is essential for reducing recidivism and achieving rehabilitation (Bonta & Andrews, 2012; Bonta, 2023). Furthermore, the rehabilitative ideal supports second-chance opportunities for individuals returning to society, whether through employment programs (D’Amico et al., 2016), educational initiatives (Bozick et al., 2018), or housing assistance (Stanley-Becker, 2022).

To be sure, the rehabilitative ideal has been subject to significant critique. One common criticism, articulated by Allen (1959), argues that an overemphasis on rehabilitation risks minimizing the importance of accountability and punishment in the criminal legal system. Some scholars draw a distinction between humane treatment and rehabilitation, asserting that institutions can uphold human dignity without necessarily aiming to transform individuals (Logan & Gaes, 1993). The most notable challenge to the rehabilitative ideal came from Martinson’s (1974) study, which questioned the effectiveness of rehabilitative programs, sparking what became known as the “nothing works” doctrine. Martinson’s research suggested that rehabilitative interventions often failed to achieve their intended outcomes. This led to widespread skepticism about the value of rehabilitation in correctional institutions. However, subsequent research has called for a balanced approach that combines treatment opportunities with accountability, recognizing that effective rehabilitation requires a focus on both individual growth and responsibility (McNeil, 2014). Despite these debates, scholars on both sides of the issue agree on one key point: the humane treatment of individuals in custody is paramount. Whether the focus is on punishment or rehabilitation, the preservation of life and wellbeing remains a critical responsibility of correctional institutions. This is reflected in the various federal and state policies aimed at protecting individuals in custody. For example, the Prison Rape Elimination Act and Texas’s Sandra Bland Act (Senate Bill 1849) are legislative efforts that reinforce the ethical imperative to safeguard the lives of incarcerated individuals.

The rehabilitative ideal continues to influence correctional practices and policies, with an overarching goal of balancing treatment with accountability. This balance is particularly relevant when considering the importance of preserving life within correctional settings. The preservation of life, after all, is both a precondition for and a product of humane treatment and effective rehabilitation. Moreover, given that the rehabilitative ideal’s emphasis on preserving life and addressing underlying factors such as substance use, it becomes crucial to examine how opioid-related deaths in correctional settings challenge these principles. The next section will explore the rise of opioid-related fatalities within correctional institutions and assess the role that both rehabilitative and punitive approaches play in mitigating or exacerbating these tragic outcomes.

### Opioid-related deaths in correctional institutions

The increasing number of drug-related deaths in correctional institutions (Carson, 2021) directly contradicts the principles of the rehabilitative ideal, particularly within the broader overdose crisis in the United States, which has been largely driven by opioids. While detection and reporting efforts in jails are lacking, considering the saturation of fentanyl in surrounding drug markets, opioid overdoses attributed to fentanyl are likely increasing in jail settings (Kaplowitz et al., 2021). Further, considerable research focuses on opioid fatalities after release; deaths occurring within correctional settings remain understudied despite these environments offering critical opportunities for intervention. Individuals leaving incarceration are especially vulnerable to opioid overdose due to the reduced tolerance developed during incarceration. Beletsky et al. (2015) found that those reentering the community are 130 times more likely to die from an overdose within the first two weeks of release compared to the general population. Joudrey et al. (2019) attributes this heightened risk to reduced respiratory tolerance, exacerbated by disruptions in medical care and substance use treatment, including medication for opioid use disorder (MOUD). Guastaferro et al. (2022) also highlight the issue of limited use of MOUD throughout the criminal legal system and how this is counterintuitive to rehabilitation. In addition, confinement conditions, such as solitary housing, further elevate these risks, with individuals placed in restrictive housing being 127% more likely to die from opioid-related overdoses upon release (Brinkley-Rubinstein et al., 2019).

Access to addiction treatment during incarceration is crucial in preventing opioid-related deaths. However, gaps in healthcare access, compounded by the challenges of returning to the community, exacerbate the risk of fatal outcomes. Individuals often return to environments lacking social support and economic stability, contributing to high relapse rates and fatalities (Binswanger et al., 2012). For instance, in North Carolina, a study funded by the Justice Community Opioid Innovation Network (JCOIN) reported a 32% increase in overdose fatalities among formerly incarcerated individuals, while rates in the general population declined (Ranapurwala et al., 2022). Disparities in outcomes are especially evident across race, sex, and geography. Black individuals experienced a 101% increase in overdose deaths between 2014 and 2018, nearly three times the rate of White individuals (Taylor et al., n.d.), while women face higher risks of opioid-related fatalities compared to men (Binswanger et al., 2013). Additionally, areas with concentrated disadvantages report disproportionately higher overdose rates (Fox et al., 2019), further complicating efforts to address the crisis. Effective treatment models, such as MOUD, reduce post-release overdose mortality by up to 80%, yet these programs are inconsistently available, particularly in rural or underserved areas (Lim et al., 2022; Howard et al., 2016). While 70% of jails report offering some form of opioid use disorder (OUD) treatment, only 38% provide reentry programs that ensure continuity of care (Scott et al., 2022). Disparities in access to MOUD also disproportionately affect Black individuals, who are more likely to rely on emergency services post-release due to inadequate healthcare access in correctional facilities (Hochstatter et al., 2021). Together, these findings underscore the urgent need for a more equitable approach to opioid treatment in correctional settings. Without immediate action, the current system will continue to exacerbate this public health crisis among incarcerated and formerly incarcerated populations.

### The broader context matters

“Their [hospitals] professional presence and capacity for offering treatment services in the community is likely to be associated with a reduced perceived need for mental health operations within the jail environment. Many jails operating in close proximity to community hospitals simply transport the most severely mentally ill to the hospitals and clinics for needed services or alternatively bring medical and mental health staff into the jail to address inmate mental health concerns” (Helms et al., 2016, p. 1051).

Within the nexus of opioid-related deaths in custody and the proposed link to rehabilitative facilities lies an important argument: the rehabilitative potential of a facility is directly tied to the area in which it operates. While individual and facility-level characteristics are significant, broader contextual factors - such as resources, accessibility, proximity, and the legal landscape - also play a critical role in shaping the health and safety of incarcerated individuals.^2^ For instance, access to emergency medical services and mental health care has been shown to foster safer custodial environments (Batastini et al., 2020; Skubby et al., 2013). Although studies specifically examining opioid deaths in custody are limited, evidence suggests that proximity to external resources, such as hospitals and mental health professionals, enhances the rehabilitative capacity of jails (Rosen et al., 2024). For example, nearby hospitals can alleviate the burden on correctional facilities by providing essential care for severe medical or mental health crises (Helms, 2016; Chari et al., 2016). Other contextual factors further underscore the importance of external infrastructure. Proximity to medical facilities is critical for managing emergencies effectively (Guagliardo, 2004; Zhong et al., 2021). In the case of a drug overdose or other medical emergency (e.g., suicide or homicide attempts), delays in accessing care can be the difference of life and death. This is also evident in community settings where scholarship shows the distance from the emergency site (e.g., a shooting, car accident, or medical emergency) to a hospital is positively associated with fatality rates (Bertoli & Grembi, 2017; Hanink et al., 2023). Moreover, limited access to public transportation isolates jails, reducing inmate visitation and external support, which can have adverse effects on mental health (Sitren et al., 2021; St. John, 2020). Scholarship indicates that pro-social support can reduce behaviors like suicide attempts or substance use among justice-involved persons (Liu & Visher, 2021; Anderson, 2018; Tillson et al., 2024). Similarly, the urbanicity of a jail’s location significantly influences access to healthcare and community resources. Urban areas generally benefit from more robust healthcare systems, social services, and support programs compared to rural regions, where resource scarcity often exacerbates existing challenges (Helms et al., 2016; Douthit et al., 2015; Pogrebin, 1982). To be sure, outside of the context of opioid-related deaths behind bars, research in the areas of education and criminal justice have factored in contextual factors as measures of rehabilitation of punitiveness (e.g., see Evans et al., 2023, on Civil War affiliations as proxies of punitiveness). These insights highlight the need to consider jails as part of a broader community infrastructure when addressing opioid-related deaths in custody. By integrating contextual factors that indicate whether a facility operates within a rehabilitative or punitive environment, this study offers a novel lens for understanding how external conditions influence opioid fatalities in U.S. jails.

### Research questions

The body of research on opioid-related fatalities in the correctional system continues to expand, detailing the factors that influence whether incarcerated individuals die from opioid use. However, there remains critical gaps in understanding the specific predictors of substance-related deaths and opioid-related deaths disaggregated from the larger grouping, within carceral settings. To be clear: (1) there are limited empirical studies examining opioid-related deaths in U.S. jails; and (2) the research team found no studies that assess both the rehabilitative aspects of a facility and its state and county conditions in relation to fatal opioid overdoses behind bars. Addressing these void is essential, as it enables a more precise allocation of strategies and resources to manage substance use in jails by considering: (a) the prevalence of opioid-related deaths in U.S. jails, and (b) how these deaths are influenced by individual, social, and systemic factors, particularly within the context of a rehabilitative or punitive locality. This study goes beyond exploring the broader issue of drug-related deaths by focusing specifically on the unique predictors of opioid-related fatalities, and in doing so, it highlights the dual role of rehabilitative and punitive factors in shaping outcomes. Rehabilitative contexts, evident by predictors such as whether Medicaid expansion was adopted, are expected to have an infrastructure for mitigating the likelihood of opioid-related deaths. Conversely, more punitive environments, characterized by punitive policies, like the legal presence of the death penalty, may exacerbate the risks of opioid fatalities. By examining these factors, the study also aims to contribute to one overarching question *do rehabilitative and punitive approaches differentially impact health outcomes in carceral settings*. To address these issues, the study is guided by the following research questions (RQs):

- RQ1 [Drug Overdoses vs. All Other Deaths]: To what extent do individual, facility, and community factors predict the likelihood of a person dying from a drug overdose as opposed to other causes of death in jail?
- RQ2 [Opioid Overdoses vs. All Other Deaths]: Is there a similar relationship for opioid-related deaths specifically?
- RQ3 [Drug Overdoses vs. Opioid Overdoses]: Are predictors of opioid-related deaths distinct from predictors of other drug-related fatalities?

By filling the current void in understanding the distinct predictors of drug-related versus opioid-specific deaths, this study aims to provide a more nuanced view of how carceral environments shape health outcomes. The emphasis on rehabilitative and punitive factors offers a critical lens for assessing the effectiveness of current strategies and for identifying potential areas for policy improvement that may go beyond the facility-level.

## Research design and methods

### Research setting

Given the unique vulnerabilities and health risks faced by individuals within the U.S. jail system, this study focuses on these facilities as critical sites for analyzing opioid-related deaths. The U.S. jail system serves as a primary interface with the criminal justice system, functioning as the initial holding facility for nearly all individuals charged with crimes, including those later transferred to prisons for longer sentences. This focus is critical due to the heightened vulnerabilities associated with the initial stages of incarceration, including high exposure to various health risks. As reported by Minton and Zeng (2021), jails experience a high turnover of individuals, underscoring the need for effective health interventions. Moreover, the initial period of incarceration is characterized by increased risks of psychological distress, suicidal ideation, and substance use relapse, which emphasizes the importance of timely and adequate healthcare services (Compton et al., 2010; Bronson et al., 2017). Jails also represent a particularly risky environment for overdoses due to the discontinuity in medications for opioid use disorder (MOUD), such as buprenorphine, which are more available in prisons. This discontinuity increases the risk of opioid overdoses both during and immediately after jail stays (Duwe, 2022; Carson, 2021).

### Data sources

To examine the risks and outcomes associated with opioid-related deaths in jails, data were collected from multiple sources, including Reuters’ death-in-custody data obtained through Freedom of Information Act (FOIA) requests, as well as publicly available datasets. FOIA requests were sent to all U.S. states, seeking individual- and facility-level data on deaths in custody from 2009 to 2019, ensuring that the ten largest county jails in each state and those with an average daily population (ADP) of 750 or more were represented. This data was merged with county- and state-level data from several publicly available sources, including county-level economic data from the American Community Survey (ACS); jail characteristics, such as security level and year of construction, retrieved from official jail websites; and spatial data, such as facility locations and proximity to highways, obtained from Google Maps. Additionally, opioid dispensary rates by county and geospatial coordinates of hospitals were retrieved from the Centers for Disease Control and Prevention, as well as the JCOIN data repository. Publicly available data on state-level policies, such as Medicaid expansion and the death penalty, were sourced from state health departments and DeathPenaltyInfo.org. The associated data files are available to share to advance the field (e.g., use for replication or merging with other data).

### Variables

This comprehensive dataset enabled an analysis of individual characteristics (e.g., age, sex, race, custody status, length of stay); punitive metrics (e.g., average daily population, presence of the death penalty, facilities built post-Title II of the 1994 Crime Bill, and historical Civil War affiliations); rehabilitative metrics (e.g., Medicaid expansion, hospital accessibility, highway and bus accessibility); key covariates (e.g., urbanicity, opioid dispensing rates, and median household income of the surrounding community); and the primary outcome of interest - cause of death while incarcerated. To be clear, variable selection is grounded in the conceptual framework outlined earlier. Variables that do not align directly with the framework but are supported by existing research are included as covariates when available in the compiled data.

The independent and individual-level variables of interest in this study are an individual’s age, race, sex, custody status, and length of stay. “Age” is a continuous measure of an individual’s age in years. “Race” includes the values of “0” for individuals who are White; “1” for individuals who are Black; “2” for individuals who are Hispanic; and “3” for individuals who are of any other race or ethnicity. “Sex” is dummy coded to indicate whether a person in custody was male (coded 0) or female (coded 1). Whether an individual was convicted of a crime or not while detained is coded “0” for not convicted and “1” for convicted. “Length of Stay” or LOS is a binary measure where the median time in days before a person died is used as the cut-off (i.e., 17 days).

Rehabilitative Variables are measured at both the facility, county, and state-levels. At the facility and county-level, the scholars include “Distance to Bus” and “Distance to Highway,” both ordinal measures in miles reflecting the proximity of the jail to the nearest highway and bus stop access point to the public – coded “0” for under a mile, “1” for one to two miles, and “2” for over two miles. Similarly coded is “Distance to Hospital” which reflects the jail distance to the nearest healthcare facility in miles. These variables assess how accessible healthcare services are and how easily persons can be accessed by members of the public (e.g., supportive family), which can be critical for medical, mental, and behavioral health interventions. At the state-level, “Medicaid Expansion” is included as a key rehabilitative metric, coded as “0” if the state has not adopted Medicaid expansion and “1” if it has. Medicaid expansion impacts access to healthcare services both during and after incarceration, making it a vital variable for analyzing health outcomes.

Punitive variables capture measures that reflect the penal environment at the facility, county, and state-levels. At the facility and county-level, “Security Level” is a categorical variable where “0” indicates minimum security, “1” indicates medium security, and “2” indicates maximum security. The highest level of security present at the facility is used for classification, reflecting the degree of punitiveness within a facility. “Overcrowded” is a dummy variable where “0” indicates that a facility is not overcrowded and “1” indicates that a facility is overcrowded. This was first calculated as the ratio of a facility’s average daily population (ADP) to its rated bed capacity, with values greater than 1.0 indicating that the facility was operating above capacity. “Crime Bill” is a binary variable reflecting whether the facility was built before or after the shift toward expanding the U.S. carceral footprint and tough on crime policies. At the state-level, “Death Penalty” is a binary measure where “0” reflects states where the death penalty is not legal, and “1” reflects states where it is legal. Additionally, “Civil War Affiliation” is coded as “0” for confederate states, “1” for unincorporated territories, “2” for union states, and “3” for border states, serving as a historical measure of punitive cultural legacy.

Covariates at the county-level include socioeconomic and demographic factors. “Urbanicity” is measured using the Center for Disease Control and Prevention’s six-level classification, with “0” for large central metros, “1” for large fringe metros, “2” for medium metros, “3” for small metros, “4” for micropolitan areas, and “5” for non-core rural areas. “Median Income” is a continuous variable which indicates a county’s median household income. Additionally, “Opioid Dispense Rate” is a county-level metric for the rate of prescription drugs dispersed. “Year” is also a continuous, numeric variable capturing the calendar year of each recorded in‑custody death (e.g., 2010, 2011…2019). This variable serves as a temporal control, allowing for an adjustment for secular changes over time or broader public health interventions that may influence the odds of opioid-related fatalities independently of other predictors. These covariates help control broader community characteristics that may influence health outcomes in jails.

The outcome variables of interest “Drug v All” measures whether a person died from a drug-related overdose while incarcerated or from another cause, with “1” reflecting that a person did and “0” reflecting that they did not die form a drug-related overdose. “Opioid v All” measures whether a person died from an opioid-related overdose while incarcerated or from another cause, with “1” reflecting that a person did and “0” reflecting that they did not die form an opioid-related overdose. “Opioid V Drug” is similarly coded, but measures whether a person died from an opioid-related overdose while incarcerated or from another drug.

### Analytical strategy

In the first analyses, multivariate logistic regression models will be employed to examine the factors associated with drug-related and opioid-related deaths in correctional facilities. Given the binary nature of these outcomes the logistic regression model is appropriate to model these data (Peng et al., 2002). This model will analyze the relationship between individual-level demographic and incarceration factors and the likelihood of a drug-related or opioid fatality – given insight into the factors that contribute to these fatalities and allowing us to assess for any difference by race, sex, and other factors that appeared in the scan of literature earlier that may impact whether someone dies from an overdose. For logistic regression, the probability of a binary outcome (e.g., whether a person dies from a drug overdose) is modeled as shown in the following equation:

logit(P(drug_death))=β0​+β1​(age)+β2​(Black)+β3​(Hispanic)+β4​(Other)+β5​(Female)+β6​(Convicted)+β7​(Length of Stay).

Next, the scholars will extend this analysis by employing a multivariate Poisson regression model with robust standard errors to examine the rate of drug-related and opioid related deaths, using an incidence rate ratio (IRR) as the key output. Poisson models are best suited for these analyses given the positively skewed and equidispersed rate outcomes (Frome et al., 1973). The rate of deaths will be modeled as a function of rehabilitative and punitive factors to assess how these characteristics contribute to drug overdose death rates, opioid overdose death rates, and overall death rates. For Poisson regression, the logarithm of the expected count of the study outcomes (e.g., drug-related death rates) is modeled as shown in the following equation:

log(λi​)=β0​+β1​(death_penalty)+β2​(civil_war_affiliation)+β3​(median_income)+β4​(urbanicity)+β5​(overcrowded)+β6​(security_level)+β7​(opioid_dispense_rate)+β8​(hospital_distance)+β9​(highway_distance)+β10​(crime_bill)+β11​(year).

Pre-analytical diagnostics (basic tests for regression assumptions) were conducted before all analyses as well as post-hoc assessments were completed (e.g., variance inflation factor checks yielded low estimates). All the statistical analyses were completed in STATA 18.

## Results

### Descriptive statistics

The average age of individuals in the sample is 44, ranging from 18 to 93 years. In terms of race, the majority are White (60.41%), followed by Black (32.22%), Hispanic (4.83%), and Other racial or ethnic groups (2.53%). The sample consists primarily of males (85.1% male), with females comprising 14.9%. Regarding custody status, 20.54% of individuals were convicted at the time of death, while 79.46% were not convicted. Length of stay (LOS) is a binary variable with a median cut-off of 17 days, where 52.19% of individuals had been incarcerated for under 17 days or less, and 47.81% were incarcerated longer than 17 days before death.

Examining rehabilitation variables, at the facility level, distance to the nearest bus stops show that 47.86% are under 1 mile in distance, 13.01% are 1 to 2 miles, and 39.13% are more than 2 miles away. In addition, distance to the nearest highway show that 63.33% are under 1 mile in distance, 20.97% are 1 to 2 miles, and 15.7% are more than 2 miles away. Moreover, distance to the nearest hospital show that 23.73% are under 1 mile in distance, 22.67% are between 1 and 2 miles in distance, and 53.60% are 2 miles or more away from a facility - representing a critical measure for assessing access to emergency healthcare services. At the state-level, Medicaid expansion is a key rehabilitative variable, with 52.14% of states adopting Medicaid expansion and 47.86% of states not expanding Medicaid.

Punitive variables capture the penal environment at both the facility, local, and state-levels. At the facility and county-level, security level is distributed as follows: 3.05% of facilities are classified as minimum security, 91.92% as medium security, and 5.04% as maximum security. Overcrowded facilities made up 43.29% of the sample. Regarding the Crime Bill, 18.86% of facilities were constructed post-1994, reflecting the shift in policies aimed at expanding the U.S. carceral system. At the state-level, death penalty laws vary, with 59.52% of states where the death penalty is legal and 40.48% where it is not. The Civil War affiliation of each state is categorized as Confederate (32.1%), Union (49.09%), Border state (7.67%), or Unincorporated territory (11.13%), serving as a historical measure of punitive legacy.

Study covariates at the county-level include urbanicity which is classified according to the CDC’s six-level categorization: large central metros (19.39%), large fringe metros (24.43%), medium metros (32.81%), small metros (15.11%), micropolitan areas (6.09%), and non-core rural areas (2.17%). Median income is a continuous measure, with $55,844.78 being the average median household income across counties. The opioid dispense rate, measured at the county level, varies from 18.6 to 240.6 per 100 persons, providing a critical context for opioid access within the broader community.

Finally, outcome variables show the proportion of individuals who died from drug-related causes while incarcerated, with 9.67% of the sample classified as such. Opioid v all other deaths further specifies opioid-related deaths, where 5% of individuals died from opioid-related causes. Lastly, Opioid v Drug identifies the proportion of drug deaths attributable to opioids, with 50.88% classified as opioid-related among all drug deaths. Table 1 provides all sample statistics. Associated rates also illustrate that the rate for opioid related deaths is 16 per 100,000 people, and for drug-related deaths is 35 per 100,000 persons. Given that all observations represent a fatal outcome, these descriptives collectively provide initial insight into the prevalence of drug-related deaths and opioid-related deaths in U.S. jails, and proportions concerning individual, rehabilitative, and punitive measures.

**Table 1:** Sample statistics

Variable (Values)	*N*	Mean (SD) or %	Min	Max
Age	1,738	44 (14)	18	93
Sex	1,738		0	1
Male (0)	1,479	85.1		
Female (1)	259	14.9		
Race/Ethnicity	1,738		0	3
White (0)	1050	60.41		
Black (1)	560	32.22		
Hispanic (2)	84	4.83		
Other Race/Ethnicity (3)	44	2.53		
Length of Stay	1,738		0	1
17 Days and Under (0)	907	52.19		
Over 17 Days (1)	831	47.81		
Custody Status	1,738		0	1
Not Convicted (0)	1,381	79.46		
Convicted (1)	357	20.54		
Distance to Bus	1,707		0	2
Under 1 Mile (0)	817	47.86		
1–2 Miles (1)	222	13.01		
2 + Miles (2)	668	39.13		
Distance to Highway	1,707		0	2
Under 1 Mile (0)	1,081	63.33		
1–2 Miles (1)	358	20.97		
2 + Miles (2)	268	15.7		
Hospital Distance	1,707		0	2
Under 1 Mile (0)	405	23.73		
1–2 Miles (1)	387	22.67		
2 + Miles (2)	915	53.6		
Medicaid Expansion	1,707		0	1
No (0)	817	47.86		
Yes (1)	890	52.14		
Facility Security Level	1,707		0	2
Minimum (0)	52	3.05		
Medium (1)	1,569	91.92		
Maximum (2)	86	5.04		
Overcrowded	1,707		0	1
Not Overcrowded (0)	968	56.71		
Overcrowded (1)	739	43.29		
Facility Construction	1,707		0	1
Before Crime Bill (0)	1,385	81.14		
After Crime Bill (1)	322	18.86		
Death Penalty	1,707		0	1
No (0)	691	40.48		
Yes (1)	1,016	59.52		
Civil War Affiliation	1,707		0	3
Confederacy (0)	548	32.1		
Unincorporated (1)	190	11.13		
Union (2)	838	49.09		
Border (3)	131	7.67		
Urbanicity	1,707		0	5
Large Central Metro (0)	331	19.39		
Large Fringe Metro (1)	417	24.43		
Medium Metro (2)	560	32.81		
Small Metro (3)	258	15.11		
Micropolitan (4)	104	6.09		
Non-core (5)	37	2.17		
Median Household Income	1,707	55,844.78 (14,024.25)	26,841	121,133
Opioid Dispense Rate	1,707	83.69 (33.85)	18.6	240.6
Drug-related Death v All Other Death			0	1
All other Death (0)	1,570	90.33		
Drug-related Death (1)	168	9.67		
Opioid-related Death v All Other Death	1,738		0	1
All other Death (0)	1,651	94.99		
Opioid-related Death (1)	87	5.01		
Opioid-related Death v Drug-related Death	171		0	1
Drug-related Death (0)	84	49.12		
Opioid-related Death (1)	87	50.88		
Drug Death Rate	1,707	35.54 (116)	0	1,785
Opioid Death Rate	1,707	16.54 (84.24)	0	1,785

### Logistic regression results

Several significant relationships appeared. Model 1 (Drug Overdose vs. All Other Deaths) show that for every additional year of age, the odds of dying from a drug overdose (compared to dying from any other cause) decrease by approximately 4% (OR = 0.96). Female individuals have 85% higher odds of dying from a drug overdose compared to male individuals (OR = 1.85). Additionally, individuals with a longer length of stay (above the median) had significantly lower odds of dying from a drug overdose - specifically, 62.6% lower odds compared to those with shorter stays (OR = 0.37). Model 2 (Opioid Overdoses vs. All Other Deaths) reveals that for each additional year of age, the odds of dying from an opioid overdose (compared to any other cause of death) decrease by approximately 4% (OR = 0.96). Finally, Model 3 (Drug Overdoses vs. Opioid Overdoses) shows that individuals who stay longer in custody are over 4.9 times more likely to die from opioid-related causes compared to other types of drugs (OR = 4.85). Table 2 provides the full results for these analyses.

**Table 2:** Logistic regression results

Variable Name	Model 1 Drug Death v All Death		Model 2 Opioid Death v All Death		Model 3 Opioid Death v Drug Death	
	OR (95% CI)	SE	OR	SE	OR	SE
Age	0.96*** (0.95–0.97)	0.01	0.96*** (0.94–0.98)	0.01	0.99 (0.96–1.03)	0.02
Race						
White (Base)						
Black	0.91 (0.63–1.30)	0.17	0.96 (0.55–1.65)	0.27	0.98 (0.39–2.49)	0.47
Hispanic	1.20 (0.56–2.60)	0.47	0.73 (0.24–2.26)	0.42	0.56 (0.13–2.41)	0.42
Other	1.10 (0.44–2.77)	0.52	1.24 (0.39–3.91)	0.78	0.80 (0.19–3.35)	0.58
Sex						
Male (Base)						
Female	1.85*** (1.29–2.65)	0.34	1.45 (0.83–2.53)	0.41	0.88 (0.44–1.73)	0.30
Custody Status						
Not Convicted (Base)						
Convicted	1.49 (0.96–2.31)	0.33	1.62 (0.97–2.70)	0.42	1.12 (0.49–2.53)	0.47
Length of Stay						
17 Days or Below (Base)						
Greater than 17 Days	0.37*** (0.26–0.54)	0.07	0.77 (0.49–1.21)	0.18	4.85*** (1.95–12.09)	2.26
Year	1.15*** (1.08–1.24)	0.04	1.16*** (1.06–1.27)	0.05	1.05 (0.92–1.19)	0.07
Constant	0.00	0.00	0.00	0.13	0.00	0.00

Note. ***P < .001. **P < .01. *P < .05.; OR = Odds Ratio; SE = Standard Error; 95% CI = 95% Confidence Interval

### Poisson regression results

Poisson regression models resulted in the identification of significant relationships. Model 4 (Drug Death Rate) indicates that individuals in small metro and micropolitan areas have 123% and 356% higher rates of drug-related deaths, respectively, compared to individuals in large central metro areas (IRRs = 2.23 and 4.56, respectively). Facility security levels show that drug death rates are 165% higher in medium-security facilities compared to minimum-security facilities (IRR = 2.65). Distance to the nearest bus stop was also a significant predictor, with facilities located more than 2 miles away from the nearest bus stop having a 66% higher rate of drug-related deaths compared to those within 1 mile (IRR = 1.66).

Examining the Opioid Death Rate (Model 5), the authors observe that individuals in large fringe metro areas have a 47% lower rate of opioid deaths compared to individuals in large central metro areas (IRR = 0.47). Results also show that facilities located more than 2 miles away from the nearest bus stop have an 82% higher rate of opioid-related deaths compared to facilities closer to public transportation access points (IRR = 1.82). Table 3 provides full results for these analyses.

**Table 3:** Poisson regression results

Variable Name	Model 4 Drug Death Rate		Model 5 Opioid Death Rate	
	IRR (95% CI)	SE	IRR	SE
Death Penalty	0.87 (0.58–1.30)	0.18	0.59 (0.30–1.16)	0.20
Civil War Affiliation				
Confederacy (Base)				
Unincorporated	0.79 (0.40–1.54)	0.27	1.14 (0.49–2.64)	0.49
Union	1.33 (0.88–2.01)	0.28	1.72 (0.88–3.37)	0.59
Border	0.74 (0.35–1.57)	0.28	1.91 (0.67–5.43)	1.02
Median Household Income	1.00 (0.00–0.00)	0.00	1.00 (1.00–1.00)	0.00
Urbanicity				
Large Central Metro (Base)				
Large Fringe Metro	0.79 (0.40–1.54)	0.27	0.47* (0.22-1.00)	0.18
Medium Metro	1.39 (0.85–2.28)	0.35	0.93 (0.44-2.00)	0.36
Small Metro	2.23** (1.30–3.81)	0.61	0.75 (0.32–1.75)	0.32
Micropolitan	4.56*** (2.41–8.63)	1.48	2.04 (0.69–6.02)	1.13
Non-core	2.44 (0.37–16.29)	2.36	3.27 (0.51–20.84)	3.09
Overcrowded				
Not Overcrowded (Base)				
Overcrowded	1.09 (0.78–1.53)	0.19	0.83 (0.46–1.48)	0.25
Facility Security Level				
Minimum (Base)				
Medium	2.65* (1.08–6.48)	1.21	3.70 (0.47–29.34)	3.91
Max	3.13 (0.95–10.35)	1.91	2.58 (0.27–24.67)	2.97
Opioid Dispense Rate	1.00 (0.99–1.01)	0.00	1.00 (1.00-1.01)	0.00
Hospital Distance				
Less than 1 Mile (Base)				
1–2 Miles	0.79 (0.50–1.23)	0.18	0.71 (0.36–1.39)	0.25
More than 2 Miles	0.77 (0.51–1.17)	0.16	1.06 (0.56–2.01)	0.35
Facility Construction				
Before Crime Bill (Base)				
After Crime Bill	1.09 (0.76–1.56)	0.20	0.87 (0.48–1.59)	0.27
Medicaid Expansion Early				
No (Base)				
Yes	1.21 (0.81–1.80)	0.21	1.18 (0.54–2.61)	0.48
Distance to Bus				
Less than 1 Mile (Base)				
1–2 Miles	1.05 (0.58–1.89)	0.32	0.90 (0.34–2.41)	0.45
More than 2 Miles	1.66** (1.16–2.36)	0.30	1.82* (1.09–3.04)	0.48
Distance to Highway				
Less than 1 Mile (Base)				
1–2 Miles	0.90 (0.59–1.37)	0.19	1.07 (0.50–2.29)	0.41
More than 2 Miles	0.86 (0.50–1.46)	0.23	1.21 (0.55–2.65)	0.48
Year	1.14*** (1.07–1.21)	0.03	1.18*** (1.08–1.30)	0.06
Constant	0.00	0.00	0.00	0.00

Note. ***P < .001. **P < .01. *P < .05.; IRR = Incidence Rate Ratio; SE = Standard Error; 95% CI = 95% Confidence Interval

### Post-Hoc examinations

Building on the exploratory nature of the study, the research team conducted additional analyses to further examine certain significant relationships found in the logistic regression models. Specifically, the scholars conducted subgroup analyses that were not originally planned, focusing on categories of key covariates that emerged as significant in the main models. Subgroups were created for sex (female and male respondents) and for length of stay, as both variables were originally discrete in nature, allowing for methodologically clear and theoretically relevant stratification. This approach enabled the research team to examine whether observed relationships with overdose mortality varied meaningfully across these distinct subpopulations. Other significant covariates (i.e., age) which was originally measured on a continuous scale, was not used as the basis for subgroup analysis to avoid imposing arbitrary thresholds or compromising statistical interpretability.

To begin, the results from the subgroup analyses of female and male populations indicate that older males were more likely than younger males to die from a drug-related cause relative to other causes of death (OR = 0.95), as shown by the odds of drug-related death increasing with age. Additionally, convicted males were 74% more likely than unconvicted males to die from a drug-related cause rather than from other causes (OR = 1.74). Across both female and male subsamples, individuals with a length of stay greater than 17 days were less likely to die from a drug-related cause relative to other causes of death. This protective effect was more pronounced in the male subsample (OR = 0.42 for males; OR = 0.19 for females), suggesting a particularly elevated risk of drug-related mortality early in the period of incarceration. Table 4 provides these results in detail.

**Table 4:** Logistic regression Results – Sex subgroup analysis

Variable Name	Model 6 Female Only Drug Death v All Death		Model 7 Male Only Drug Death v All Death	
	OR (95% CI)	SE	OR (95% CI)	SE
Age	0.98 (0.96–1.01)	0.01	0.95*** (0.94–0.97)	0.01
Race				
White (Base)				
Black	0.44 (0.16–1.20)	0.23	1.04 (0.70–1.59)	0.21
Hispanic	0.93 (0.19–4.53)	0.75	1.31 (0.56–3.06)	0.57
Other	1.17 (0.23–6.03)	0.98	0.98 (0.30–3.15)	0.58
Custody Status				
Not Convicted (Base)				
Convicted	1.05 (0.46–2.38)	0.61	1.74* (1.05–2.89)	0.45
Length of Stay				
17 Days or Below (Base)				
Greater than 17 Days	0.19** (0.07–0.56)	0.11	0.42*** (0.27–0.63)	0.09
Year	1.12 (0.98–1.28)	0.08	1.17*** (1.08–1.26)	0.05
Constant	1.12	0.08	0.00	0.00

Note. ***P < .001. **P < .01. *P < .05.; OR = Odds Ratio; SE = Standard Error; 95% CI = 95% Confidence Interval

Next, a subgroup analyses based on LOS indicates that older people across both sub samples of persons held in a facility over 17 days or under 17 days are less likely to dies from a drug related death relative to younger persons (OR = 0.93 for the over 17 days subgroup and OR = 0.97 for the under 17 days subgroup). Additionally, females relative to males who were held in a facility under 17 days were 1.28% times more likely to die from a drug related death relative to all other causes of death. Lastly, person who were convicted relative to persons who were not convicted and held in facilities over 17 days were 1.54 times more likely to die from a drug-related overdose relative to all other forms of death (OR = 2.54). A closer examination into where these opioid related facilities cluster around intake, the scholars observe that 35% of opioid related deaths and 54% of other drug-related deaths occur within 24 h. Tables 5 and 6 provide these results in detail.

**Table 5:** Logistic regression Results – Length of stay subgroup analysis

Variable Name	Model 8 – Over 17 Days Drug Death v All Death		Model 9 – Under 17 Days Drug Death v All Death	
	OR (95% CI)	SE	OR	SE
Age	0.93*** (0.91–0.95)	0.01	0.97*** (0. 96-0.99)	0.01
Race				
White (Base)				
Black	0.89 (0.41–1.90)	0.34	0.93 (0.61–1.44)	0.21
Hispanic	0.61 (0.11–3.26)	0.52	1.64 (0.70–3.87)	0.72
Other	2.01 (0.61–6.62)	1.22	0.53 (0.14–2.07)	0.37
Sex				
Male (Base)				
Female	0.78 (0.25–2.42)	0.45	2.28*** (1.56–3.34)	0.44
Custody Status				
Not Convicted (Base)				
Convicted	2.54** (1.31–4.95)	0.86	1.15 (0.65–2.03)	0.33
Year	1.15* (1.01–1.32)	0.08	1.16*** (1.07–1.25)	0.05
Constant	0.00	0.00	0.00	0.00

Note. ***P < .001. **P < .01. *P < .05.; OR = Odds Ratio; SE = Standard Error; 95% CI = 95% Confidence Interval

**Table 6:** Length of stay (LOS) for opioid and drug deaths

	Percentage of Opioid Deaths	Percentage of Drug Deaths
Day Zero	12.79	28.57
One day or under	34.88	54.76
Two days or under	43.02	64.29
One week or under	60.47	78.57

## Discussion

Overall, the main models and post-hoc examinations provide a multidimensional view of drug- and opioid-related deaths in jail custody. In response to RQ1 and RQ2, the logistic regression models converge on age and length of stay as significant individual-level predictors of both drug and opioid-related deaths. Younger individuals are consistently at higher risk of overdose mortality across both models. For drug-related deaths (Model 1), shorter lengths of stay are associated with increased odds of death, suggesting heightened vulnerability during the earliest stages of incarceration. This may be due to withdrawal symptoms, lack of immediate care, or unmanaged substance use disorders at intake - underscoring critical gaps in screening and early intervention. For opioid-specific deaths (Model 2), age remains significant, but Model 3 (Drug vs. Opioid Deaths) reveals that individuals with longer stays are significantly more likely to die from opioids than from other drugs (OR = 4.85). While drug-related deaths overall are more likely to occur early in incarceration, opioid-specific deaths appear to become more prominent among individuals held for longer durations. This suggests that prolonged incarceration may exacerbate risk for opioid overdose specifically - potentially due to deteriorating health, gaps in sustained treatment for opioid use disorder, or cumulative stress and trauma during detention (RQ3). The post-hoc subgroup analyses reinforce and extend these findings. A large share of overdose deaths occur shortly after incarceration: 54% of non-opioid drug deaths and 35% of opioid deaths occurred within 24 h. These descriptive patterns align with the main model findings that shorter stays correlate with higher risk, particularly for non-opioid drug overdoses. Additionally, the subgroup analyses reveal that convicted individuals, particularly those held longer than 17 days, have significantly higher odds of dying from drug-related causes relative to unconvicted individuals (OR = 2.54). This may reflect not only harsher conditions or reduced access to care but also longer exposure to institutional environments, as convicted individuals typically remain in custody for extended periods. Sex emerges as another key factor in drug-related mortality (Model 1), with female individuals having 85% higher odds of dying from a drug overdose compared to males. Post-hoc results indicate that this effect is concentrated among women held fewer than 17 days, which may reflect unaddressed health needs or a lack of gender-responsive programming at intake. Notably, sex is not a significant predictor in the opioid-specific models (Models 2 and 3), suggesting that its impact may be more pronounced in the context of other substances (RQ2 and RQ3).

Turning to the Poisson models, which address death rates at the facility and community levels, the results reveal important contextual factors tied to geography, facility characteristics, and access to services (RQ1 and RQ2). Drug-related death rates are substantially higher in small metro and micropolitan areas, with 123% and 356% increases respectively, compared to large central metro areas (Model 4). For opioid deaths (Model 5), individuals in large fringe metro areas experience 47% lower death rates than those in large central metros, confirming geographic variation in exposure, health system capacity, or resource availability. Facility security level and transportation access also differentiate risk. Medium-security facilities are associated with significantly higher rates of drug-related deaths compared to minimum-security facilities (IRR = 2.65), implying that more punitive or restrictive institutional environments may exacerbate risk. However, this pattern does not hold for opioid-specific deaths, where facility security level is not a significant factor. In contrast, access to public transportation is significantly associated with overdose rates across both models. Facilities located more than two miles from the nearest bus stop exhibit a 66% higher drug death rate and an 82% higher opioid death rate, respectively, than facilities closer to public transit. This suggests that geographic isolation may limit access to external rehabilitative services, such as opioid use disorder (OUD) treatment or emergency medical care, reinforcing the importance of integrated health and transportation planning in jail contexts.

### Implications for practice and policy

Combined with preexisting literature, this study highlights the critical need for comprehensive reform in addressing drug- and opioid-related deaths within correctional facilities. Early intervention and continuous rehabilitative care are paramount in mitigating mortality rates, especially among younger individuals in custody. To effectively reduce overdose risks, it is imperative for jails to implement robust substance use disorder screenings upon admission, ensuring that treatment begins immediately. Tailored interventions, such as education, counseling, medication, and behavioral therapies following the Risk-Needs-Responsivity (RNR) model, are necessary to meet the diverse needs of incarcerated individuals. Attention must be paid to women in custody, who are often underserved by gender-neutral programs that fail to address their specific healthcare needs, such as mood disorders, PTSD, and trauma (Bahji et al., 2019; Khan et al., 2013a, b). The finding that women face significantly higher odds of dying from drug overdoses while in custody supports the urgent need for correctional systems to move beyond one-size-fits-all approaches (Lutgen-Nieves & Petty, 2024). Instead, facilities should adopt healthcare models that are responsive to women’s distinct psychosocial and behavioral health needs (Covington, 2008). At a minimum, this includes trauma history screening, access to evidence-based mental health and substance use treatment, and continuity of care upon release.

Moreover, supervised detoxification and regular health assessments by trained medical staff are critical, particularly during the first 17 days of incarceration, a period identified as crucial for preventing drug-related deaths. Notably, this study is one of the few to disaggregate opioid-specific mortality and examine when these deaths occur. While many drug-related deaths occur early - especially within the first 24 to 72 h - opioid-related deaths show a more extended pattern, including among individuals held longer in custody. This distinction suggests that early intervention alone is insufficient; correctional systems must also ensure sustained, long-term access to care throughout the incarceration period to effectively mitigate opioid-related harm. The lack of adequate screening and intervention is evident, with only 63% of jails screening for opioid use disorder at admission, 54% providing withdrawal medication, and just 29% offering overdose education (Maruschak et al., 2023; Widra, 2024). Effective overdose intervention and prevention in jails requires evidence-based public health responses. While our study did not directly assess access to Medication for Opioid Use Disorder (MOUD) or opioid overdose education and naloxone distribution (OEND), these practices have demonstrated effectiveness in reducing overdose risk in correctional settings (Macmadu et al., 2020). MOUD - including methadone, buprenorphine, and naltrexone - can reduce cravings, alleviate withdrawal symptoms, and lower fatal overdose rates (Bird et al., 2016; Huxley-Reicher et al., 2018; Moore et al., 2019). Similarly, overdose education and naloxone distribution programs have been shown to reduce mortality and are essential components of comprehensive prevention efforts (Bennett & Holloway, 2012; Bird et al., 2016; Horsburgh & McAuley, 2018). Although not directly examined in our analysis (see limitations section), these interventions represent promising strategies that align with broader public health goals and may complement the types of jail-based overdose prevention efforts needed to reduce overdose fatalities.

In addition to evidence-based medical interventions, addressing logistical barriers is crucial. Limited transportation, particularly in rural or smaller facilities, often delays emergency care. Telemedicine offers a viable alternative, providing immediate access to healthcare professionals and improving the management of at-risk individuals through consistent follow-ups. Together, these reforms necessitate a collective shift in the institutional culture of correctional facilities. Moving away from a punitive model and embracing a rehabilitative, health-centric approach is essential in managing substance use disorders and preventing overdose deaths. Addressing these complex issues requires systemic changes that prioritize the health and well-being of incarcerated individuals while implementing evidence-based preventive measures that reduce the overall risk of fatal drug and opioid overdoses.

### Limitations and future research

The study has several key limitations that suggest important directions for future research. First, the sample is restricted to instances of death in custody, which provides a limited perspective. While the absence of data on non-fatal cases constrains comparisons, analyzing the characteristics and circumstances associated with opioid-related deaths - particularly in contrast to other drug-related deaths, still provides actionable insights for harm reduction and prevention strategies in correctional settings. Second, the reliance on official correctional records introduces a potential discrepancy, especially in cases where the recorded cause of death may not fully align with the findings of medical professionals or post-mortem analyses. To improve the reliability of the cause of death data, future studies should incorporate a dual-sourcing approach by comparing official correctional records with medical reports or external reviews. This cross-referencing could help ensure that recorded causes of death more accurately reflect the actual medical circumstances. Third, the distance to public transit variables embody a theoretical tension. On one hand, facilities located farther from bus stops and highways may benefit from increased pro‑social support through visitations. However, research also indicates that negative or contentious interactions can exacerbate risks such as suicide attempts and substance use among incarcerated individuals (Mowen & Visher, 2015; Clark et al., 2016). On the other hand, proximity to transit networks and major roads may facilitate the inflow of illicit substances. Both staff and visitors serve as vectors for contraband entering correctional settings (Bucerius et al., 2023; Austin et al., 2023; Peterson et al., 2023), and jails closer to these access points are arguably more vulnerable to drug smuggling. Variations in local drug‑market activity - including differences in volume, substance types, and potency - can further shape the facility’s risk environment and affect individuals cycling between incarceration and the community. Fourth, limited data availability creates the potential for omitted variable bias given that addition of other key factors, like facility management (Harney, 2024; St. John, 2023), design features (Scott et al., 2018; Moran et al., 2023; St. John et al., 2019), staffing levels (Bardwell et al., 2018), and environmental conditions (Moran et al., 2024) may help explain whether persons die or live while under correctional custody. Further, the dataset does not include direct measures such as substance use assessment scores, risk/need profiles, or detailed histories of opioid and other drug use. Despite recommendations from the Substance Abuse and Mental Health Services Administration and the National Commission on Correctional Health Care (NCCHC, 2002; SAMSHA, 2015), substance use screening remains inadequate in jail settings (Bunting et al., 2023; Taxman et al., 2007). Without these measures, we cannot distinguish between individuals at the time of their incarceration to understand those who were actively engaged in drug use, those who had a history of use but were in recovery, and those who may never have used drugs. This means that comparisons could include individuals with vastly different risk profiles, potentially even comparing overdose deaths among those with a history of substance use to those with no previous drug use history. Future research should prioritize screening efforts and access to more granular data on substance use history in jail settings to better contextualize outcomes and refine risk stratification. Notably, as aforementioned, metrics for MAT and MOUD are key in further understanding this relationship behind bars – a metric that this study currently could not access for the county jails across time for the large sample.

To be sure, there are additional points where future research should build on current findings. First, the surrounding context in which correctional facilities operate is ripe for further inquiry - particularly the extent to which non-carceral policies and community-level practices have spillover effects that mitigate overdoses inside jails and prisons. Similarly, a deeper exploration into other drugs and specific opioid types could enhance an understanding of the circumstances and substances most associated with fatal outcomes, as observed in community-based studies (Perdue et al., 2022, 2024; Nowotny et al., 2017). Future research should also investigate how opioids and other substances enter correctional facilities, especially considering findings that nearly half of opioid-related deaths occurred among individuals incarcerated for more than 17 days. This raises important questions about institutional vulnerabilities - including staff misconduct, deficiencies in intake or visitation screening, and generally permissive supervision environments in which prescribed medications are mismanaged and illicit substances are more easily introduced into facilities (Bucerius et al., 2023; Novisky et al., 2022; Austin et al., 2023). Second, future research should include data on non-fatal overdoses to investigate what factors differentiate non-fatal vs. fatal incidents in correctional facilities. This would allow for a more nuanced understanding of the counterfactual and support the development of more precisely targeted interventions. Finally, future research would benefit from a mixed-methods approach that incorporates qualitative interviews or case studies. A qualitative component could illuminate the underlying mechanisms and lived experiences that contribute to observed statistical patterns, offering a richer understanding of both institutional practices and personal trajectories surrounding drug- and opioid-related mortality. Taken together, these approaches combined would provide a more holistic view of both life and death in the context of opioid use in jails and prisons, leading to more effective interventions aimed at reducing mortality.

## Data Availability

No datasets were generated or analysed during the current study.

## Footnotes

1 To be sure, the term rehabilitation can vary in meaning based on geography, discipline, and an array of factors (Wade, 2021).

2 State-level legislation and public health policies (e.g., Medicaid expansion) shape the fiscal, regulatory, and normative environment within which counties operate. Although county governments manage the day-to-day operations of jails, they do so within broader state-level constraints and opportunities. For example, Medicaid expansion has been shown to increase healthcare access for the public (Li, 2014) and among justice-involved populations, reducing reliance on self-medication and untreated pain that can contribute to opioid misuse or overdose (Choi et al., 2018; Albertson et al., 2020). States that expand Medicaid often reflect a more rehabilitative policy orientation, which may influence local correctional practices and service provision through normative signaling (Fry et al., 2020). For instance, Cook County Jail implemented a Medicaid enrollment initiative to enroll eligible individuals in Medicaid at the point of intake, aiming to promote healthcare continuity as a preventive measure (Riedel et al., 2016). Moreover, state-level decisions affect county capacity through fiscal transfers and regulatory structures (Shi et al., 2018). Thus, even when substantial within-state variation exists, state policies function as meaningful structural forces that shape local implementation, resourcing, and strategic orientation.
