# Correction: U.S. Jails and fatal drug overdoses: patterns, predictors and the role of rehabilitative contexts

**DOI:** 10.1186/s40352-025-00387-9

**Published:** 2025-11-28

**Authors:** Victor St. John, Tasha Perdue, Jason Szkola, Mijin Kim, Katharine McGrath, Noa Glover, Josh Sugino

**Affiliations:** 1 The Ohio State University, Columbus, USA; 2 Rowan University, Glassboro, USA; 3 Independent Researcher, Chicago, USA; 4 Saint Louis University, St Louis, USA; 5 La Canada High School, La Canada Flintridge, USA; 6 Illinois State University, Normal, IL USA

**Correction: Health Justice 13, 56 (2025)**

**https://doi.org/10.1186/s40352-025-00365-1**

Following the publication of the original article (St. John et al., 2025), the author “Mijin Kim” at ‘Illinois State University, Illinois, USA’ was inadvertently omitted from the author list.

The correct author list is given below:

Victor St. John^1^*, Tasha Perdue^1^, Jason Szkola^2^, Mijin Kim^6^, Katharine McGrath^3^, Noa Glover^4^ and Josh Sugino^5^.

Old author list:

Victor St. John^1^*, Tasha Perdue^1^, Jason Szkola^2^, Katharine McGrath^3^, Noa Glover^4^ and Josh Sugino^5^.

This has been corrected above and the original article (St. John et al., 2025) has been updated.
